# The lymphocyte levels of Hashimoto thyroiditis patients were significantly lower than that of healthy population

**DOI:** 10.3389/fendo.2025.1472856

**Published:** 2025-01-27

**Authors:** Hui Xue, Ruyi Xu

**Affiliations:** ^1^ Department of Endocrinology, Suqian First Hospital, Suqian, China; ^2^ Department of Nephrology, Suqian First Hospital, Suqian, China

**Keywords:** Hashimoto thyroiditis, lymphocyte count, lymphocyte percentage, neutrophil count, lymphocyte migration

## Abstract

**Background and purpose:**

Hashimoto thyroiditis (HT) is an autoimmune disease without infectivity. We compared the differences of blood lymphocytes levels between HT patients and healthy people.

**Patients and methods:**

This study included a total of 84 HT patients whose thyroid function was normal and 60 HT patients with abnormal thyroid function. A corresponding number of medical examination population in our hospital were randomly selected as the control groups. White blood cell count, neutrophil count, neutrophil percentage, lymphocyte count, and lymphocyte percentage were compared between HT patients and healthy population. The correlations between TSH, FT4 and above parameters were further tested.

**Results:**

We found significant differences between HT with normal thyroid function group and control group in lymphocyte count (*P*<0.001), lymphocyte percentage (*P*<0.001) and neutrophil percentage (*P*<0.001), but no differences in sex (*P*=0.134), age (*P*=0.200), white blood cell count (*P*=0.315) and neutrophil count (*P*=0.790). Significant differences were observed in neutrophil count (*P*=0.032), neutrophil percentage (*P*=0.010), lymphocyte count (*P*=0.010) and lymphocyte percentage (*P*<0.001) between HT with abnormal thyroid function group and control group, but not in sex (*P*=0.769), age (*P*=0.060) and white blood cell count (*P*=0.156) between the two groups. There were significant differences in white blood cell count (*P*=0.009) and neutrophil count (*P*=0.032) between HT patients in the normal thyroid function group and HT patients in the abnormal thyroid function group. Neither FT4 nor TSH was associated with lymphocyte levels or neutrophil levels.

**Conclusions:**

The lymphocyte levels in HT patients were significantly lower than healthy population. The neutrophil count in HT patients with regular thyroid function was lower than those in abnormal thyroid function HT patients.

## 1 Introduction

Hashimoto thyroiditis (HT), also known as chronic lymphocytic thyroiditis, is characterized by painless, diffuse goiter and the presence of high titer autoantibodies in the serum, and some patients will eventually develop hypothyroidism. In recent years, the prevalence and incidence of HT have been increasing. In China, the rate of thyroid autoimmune antibody elevation is about 14.19% (1) and women aged 30 to 50 are the highest incidence group. HT is a non-infectious autoimmune disease with unknown etiology and interaction between genetic and autoimmune factors. Based on the interactions of environmental factors and genetic background, the autoimmune manifestations of HT occur, causing an imbalance between the self-tolerance mechanisms maintained by regulatory T lymphocytes and B lymphocytes (2–4). The main cause of HT is the activation of cellular and humoral immune responses against autoantigens. The main thyroid antigens that induce antibody-mediated responses are thyroglobulin (Tg), thyroid peroxidase antigen (TPO) and thyroid stimulating hormone receptor (TSHR) (5). Thus, in patients with HT, changes in thyroid hormone levels and metabolism can be observed, leading to corresponding clinical symptoms. About 20-30% of HT patients may develop hypothyroidism (6–8). Thyroid lymphocyte infiltration (especially T cell infiltration) is the main feature of HT. The thyroid is gradually replaced by lymphocytes, which can lead to fibrosis and atrophy of thyroid cells (6). Currently, the diagnosis of HT is determined through clinical manifestations, thyroid ultrasound results and the detection of serum anti-thyroid antigen antibodies (anti-thyroid peroxidase antibodies and thyroglobulin antibodies) (7).

It is lymphocyte migration that is the mechanism causing lymphocyte infiltration in thyroid tissue of HT patients. Lymphocyte migration, being a homing mode of lymphocyte, involves a series of molecules in the specific migration of lymphocytes from blood to various tissues and organs. Studies have verified that lymphocyte migration is also implicated in the pathogenesis of other diseases, such as inflammatory bowel disease, rheumatoid arthritis and systemic lupus erythematosus (9–13). Whether the migration of a large number of lymphocytes in the blood to the thyroid tissue due to lymphocyte migration leads to a decrease in the count and percentage of lymphocyte in the blood and whether there are differences in blood lymphocyte count and percentage between HT patients with normal thyroid function and HT patients with hypothyroidism remain to be determined. Consequently, this study aimed to assess the differences in blood lymphocyte levels between HT patients and healthy population.

## 2 Methods

### 2.1 Ethical considerations

The research adhered to the Declaration of Helsinki and the local Ethics Committee approved the study.

### 2.2 Patients and study design

The study encompassed 144 HT patients. HT patients who met the diagnostic criteria and didnot undergone thyroid surgery were included. The following conditions are excluded: (1) HT patients with blood system diseases,tumor or infection, (2) HT patients who declined blood tests, (3) prior treatment with corticosteroids, (4) patients with hyperthyroidism. When the patients were first admitted to the hospital, their gender, age were recorded. On the second day of being hospitalized, venous blood was taken to examine leukocyte count, lymphocyte count, neutrophil count, lymphocyte percentage and neutrophil percentage, thyroid function. Blood routine and thyroid function were detected automatically by machine. Hypothyroidism refers to elevated thyroid stimulating hormone (TSH) and decreased free thyroid hormone 4 (FT4), and subclinical hypothyroidism refers to elevated TSH and normal FT4 (14, 15).

To compared the differences of leukocyte count, lymphocyte count, neutrophil count, lymphocyte percentage and neutrophil percentage between HT patients and healthy people, 84 medical examination population in our hospital were randomly chosen as the control group. Their corresponding indicators were also recorded.

Firstly, the differences in white blood cell count, neutrophil count, neutrophil percentage, lymphocyte count, lymphocyte percentage between HT patients with abnormal thyroid function, HT patients with normal thyroid function, and healthy controls were compared. Abnormal thyroid function included hypothyroidism (elevated TSH and decreased FT4) and subclinical hypothyroidism (elevated TSH and normal FT4). Normal thyroid function meant that FT4, FT3 and TSH are normal. If there was a difference, then the correlations between TSH, FT4 and these different parameters were further tested.

### 2.3 Statistical analysis

Descriptive data were shown as median (interquartile) or percentage. The normality of all numerical variables was examined. For nonparametric tests, the Mann-whitney U test was used, and for categorical variables, the Chi-square test or Fisher's exact test was applied. The correlation among the data was calculated by Pearson correlation analysis or Spearman rank correlation analysis. SPSS 21.0 software was utilized for statistical analysis, and a P-value less than 0.05 indicated statistical significance.

## 3 Results

### 3.1 The lymphocyte levels in the HT patient group were markedly decreased compared to those in the control group

According to thyroid function, HT patients were divided into normal thyroid function group and abnormal thyroid function group.60 HT patients with irregular thyroid function as well as 84 HT patients with regular thyroid function were present. We found significant differences between HT with normal thyroid function group and control group in lymphocyte count (*P*<0.001), lymphocyte percentage (*P*<0.001) and neutrophil percentage (*P*<0.001), but no differences in sex (*P*=0.134), age (*P*=0.200), white blood cell count (*P*=0.315) and neutrophil count (*P*=0.790). Significant differences were observed in neutrophil count (*P*=0.032), neutrophil percentage (*P*=0.010), lymphocyte count (*P*=0.010) and lymphocyte percentage (*P*<0.001) between HT with abnormal thyroid function group and control group, but not in sex (*P*=0.769), age (*P*=0.060) and white blood cell count (*P*=0.156) between the two groups. Both lymphocyte count and percentage in HT patients group were significantly lower than that in control group, whether thyroid function is normal or not (**Table 1**).

**Table 1:** Analysis of the differences between HT with normal thyroid function group and Control group, HT with abnormal thyroid function group and Control group.

	HT with normal thyroid function group, n=84	Control group, n=84	P-value	HT with abnormal thyroid function group, n=60	Control group, n=60	P-value
Sex:female, n (%)	78 (92.86%)	72 (85.71%)	0.134	54 (90.00%)	53 (88.33%)	0.769
Age (years): M (QR)	39.00 (31.00-51.00)	36.50 (23.25-48.00)	0.200	41.00 (29.00-53.00)	35.5.00 (25.25-40.60)	0.060
White blood cell: M (QR), ×10^9^/L	5.80 (4.50-6.50)	5.90 (4.90-6.90)	0.315	6.35 (5.21-7.63)	6.00 (4.90-6.98)	0.156
Lymphocyte count: M (QR), ×10^9^/L	1.70 (1.30-2.10)	2.10 (1.70-2.50)	<0.001	1.65 (1.30-2.17)	2.05 (1.70-2.50)	0.010
Lymphocyte percentage: %	30.20 (20.20-35.60)	35.10 (30.70-43.00)	<0.001	29.20 (19.20-36.70)	35.10 (30.45-40.20)	<0.001
Neutrophil count: M (QR), ×10^9^/L	3.30 (2.50-4.20)	3.30 (2.60-4.10)	0.790	3.79 (2.82-5.14)	3.35 (2.60-4.28)	0.032
Neutrophil percentage: %	60.10 (50.40-65.20)	55.25 (51.13-59.40)	<0.001	60.25 (50.65-70.57)	56.00 (51.10-59.38)	0.010

M, median; QR, Quartile Range; HT, Hashimoto thyroiditis.

### 3.2 Analysis of the differences between HT with regular thyroid function group and HT with irregular thyroid function group

No statistically significant differences were found in sex (*P*=0.686), age (*P*=0.843), lymphocyte count (*P*=0.854), lymphocyte percentage (*P*=0.107) and neutrophil percentage (*P*=0.463) when comparing the two groups. However, significant differences existed in white blood cell count (*P*=0.009) and neutrophil count (*P*=0.032) between the two groups. Neutrophil count in HT with normal thyroid function group was significantly lower than HT with abnormal thyroid function group (**Table 2**).

**Table 2:** Analysis of the differences between HT with normal thyroid function group and HT with abnormal thyroid function group.

	HT with normal thyroid function group, n=84	HT with abnormal thyroid function group, n=60	P-value
Sex:female, n (%)	78 (92.86%)	54 (90.00%)	0.686
Age (years): M (QR)	39.00 (31.00-51.00)	41.00 (29.00-53.00)	0.843
White blood cell: M (QR), ×10^9^/L	5.80 (4.50-6.50)	6.35 (5.21-7.63)	0.009
Lymphocyte count: M (QR), ×10^9^/L	1.70 (1.30-2.10)	1.65 (1.30-2.17)	0.854
Lymphocyte percentage: %	30.20 (20.20-35.60)	29.20 (19.20-36.70)	0.107
Neutrophil count: M (QR), ×10^9^/L	3.30 (2.50-4.20)	3.79 (2.82-5.14)	0.032
Neutrophil percentage: %	60.10 (50.40-65.20)	60.25 (50.65-70.57)	0.463

M, median; QR, Quartile Range; HT, Hashimoto thyroiditis.

### 3.3 Correlations between FT4, TSH and neutrophil count, neutrophil percentage, lymphocyte count, lymphocyte percentage in the 144 HT patients

**Figure 1:**
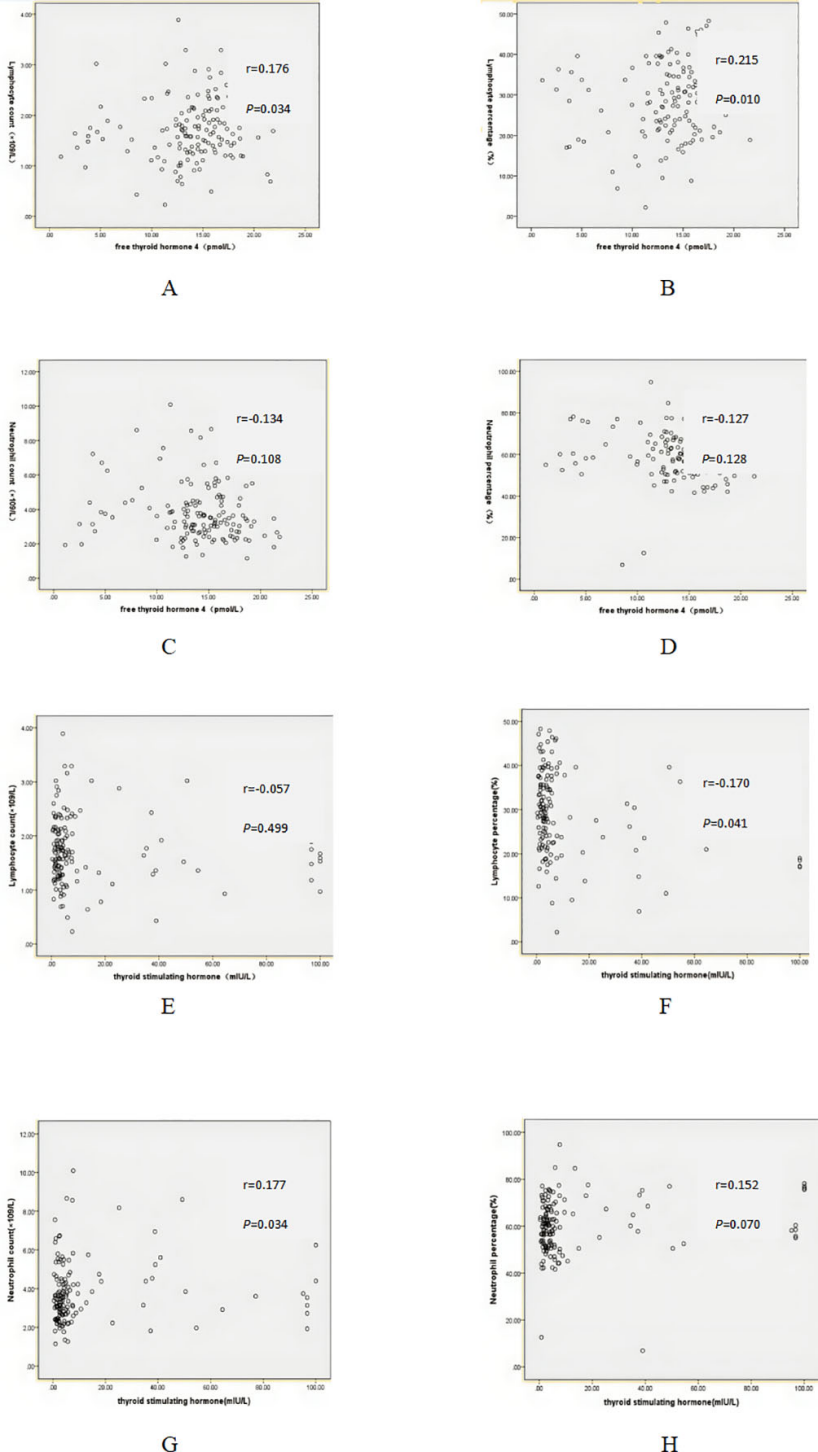
Correlation between free thyroid hormone 4 (FT4), thyroid stimulating hormone (TSH) and serum markers. **(A)** Correlation between FT4 and lymphocyte count. **(B)** Correlation between FT4 and lymphocyte percentage. **(C)** Correlation between FT4 and neutrophil count. **(D)** Correlation between FT4 and neutrophil percentage. **(E)** Correlation between TSH and lymphocyte count. **(F)** Correlation between TSH and lymphocyte percentage. **(G)** Correlation between TSH and neutrophil count. **(H)** Correlation between TSH and neutrophil percentage.

According to **Table 1**, no significant differences was observed in lymphocyte count, neutrophil count, lymphocyte percentage and neutrophil percentage between HT patients and control group. In this study, there were no linear correlations between FT4 and lymphocyte count (r=0.176, *P*=0.034), lymphocyte percentage (r=0.215, *P*=0.010), neutrophil count (r=-0.134, *P*=0.108), neutrophil percentage (r=-0.127, *P*=0.128). Similarly, there were no linear correlations between TSH and lymphocyte count (r=-0.057, *P*=0.499), lymphocyte percentage (r=-0.170, *P*=0.041), neutrophil count (r=0.177, *P*=0.034, neutrophil percentage (r=0.152, *P*=0.070) (**Figure 1**).

## 4 Discussion

HT is an autoimmune disease in which the destruction of the thyroid is the result of lymphocyte infiltration. It affects approximately 160 million people worldwide, and women are 4-10 times more susceptible than men (7, 16). In HT, a cellular immune response with high inflammatory load and apoptosis occurs, resulting in tissue destruction and thyroid dysfunction. Generally, it is thought that HT is the outcome of multiple environmental factors causing immune dysfunction on the basis of genetic predisposition. Nevertheless, the molecular mechanism by which immune dysfunction gives rise to the destruction of thyroid tissue still remains unclear (17–20). Susceptibility genes associated with HT include PTPN22, HLA-DR3, FOXP3, CD40, IL-2Rα and CTLA-4 (20–23).

The massive infiltration of lymphoid cells in the thyroid tissue of HT patients may be due to lymphocyte migration. Through a series of molecular mediations, lymphocyte migration from blood to thyroid tissue leads to lymphocyte infiltration in thyroid tissue. Studies have shown that Th1 cytokines such as interleukin-1, interleukin-6 and tumor necrosis factor- alfa (TNF-α) can mediate the infiltration and accumulation of antigen-producing B lymphocytes, cytotoxic T cells, and macrophages into thyroid tissue (24, 25). Macrophage migration inhibitory factor (MIF) can promote the production of inflammatory Th1 cytokines, including IL-2, TNF-α and IL-6 (26). A study published in 2014 showed that MIF levels were significantly higher in HT patients than in healthy people and were associated with hypothyroidism (27). This result indirectly reflected that lymphocyte migration may be involved in the pathogenesis of HT. In addition, many studies have confirmed that lymphocyte migration plays an important role in the pathogenesis of IBD (28, 29). Studies also confirmed the existence of lymphocyte infiltration in exocrine gland tissue of Sjogren's syndrome (SS), which leads to the injury of SS exocrine gland (30–32). As lymphocyte migrated from the blood to the thyroid tissue, we expected that the levels of lymphocyte in the blood of HT patients may be lower than that of healthy population. Therefore, 84 HT patients with regular thyroid function and 60 HT patients with irregular thyroid function were included to compare the differences of blood lymphocytes levels between the two groups and healthy population. The results indicated that although the total leukocyte count in HT patients had no significant difference compared to that of the healthy population, the lymphocyte count and percentage were notably lower than those of the healthy population, while neutrophil percentage was significantly higher than that of healthy population. A study published in 2020 showed that neutrophil/lymphocyte ratio was statistically higher than healthy population, a result that is consistent with this study (33). In addition, this study also found that the neutrophil count and white blood cell count in HT patients with abnormal thyroid function were significantly higher than those with normal thyroid function. A study showed that over activation and over recruitment of neutrophils at the site of inflammation are thought to contribute to the pathogenesis and progression of IBD (34). The oxidative stress damage caused by the production of reactive oxygen species is involved in the pathogenesis and progression of IBD, and neutrophil cells are an important source of reactive oxygen species. In the inflammatory microenvironment, reactive oxygen species can damage biological macromolecules such as deoxyribonucleic acid, proteins and lipids, thus causing tissue cell damage. Given that the neutrophil count in HT patients with abnormal thyroid function is considerably higher than that in HT patients with normal thyroid function, could it be that the activation of neutrophils aggravates the destruction of thyroid tissue, thereby resulting in hypothyroidism? The mechanism of this remains to be further studied. This study also studied whether FT4 and TSH were correlated with lymphocyte and neutrophil levels, but the results showed that there was no linear correlation. The reason may be that neutrophils only initiate the destruction of thyroid tissue, but not associated with severity. The severity of thyroid tissue destruction may be due to other factors. Just as the level of serum amylase can only be used to diagnose acute pancreatitis, and does not reflect the severity of acute pancreatitis.

This study has certain drawbacks. First, in this study, HT patients with hypothyroidism and subclinical hypothyroidism were collectively referred to as thyroid dysfunction, and were not analyzed separately. Secondly, while this study demonstrated that the neutrophil count in HT patients with irregular thyroid function exceeded that in HT patients with regular thyroid function, it unfortunately did not further dig into the possible mechanism underlying this phenomenon. Specifically, the sample size within this study was relatively limited and it was only a single-center investigation. Consequently, it is anticipated that more comprehensive studies will be carried out in the future, incorporating a larger number of patients to enhance the validity and generalizability of the findings. Whether monitoring lymphocyte levels can be used in the diagnosis and treatment of HT and whether neutrophil activation causes hypothyroidism remain to be investigated.

To sum up, the findings of this study possess significant clinical importance. The lymphocyte levels in HT patients were significantly lower than normal, and the neutrophil counts in abnormal thyroid function HT patients were higher than those in normal thyroid function HT patients.

## Data Availability

The raw data supporting the conclusions of this article will be made available by the authors, without undue reservation.
